# Putting habit into practice, and practice into habit: a process evaluation and exploration of the acceptability of a habit-based dietary behaviour change intervention

**DOI:** 10.1186/s12966-014-0135-7

**Published:** 2014-10-30

**Authors:** Benjamin Gardner, Kate Sheals, Jane Wardle, Laura McGowan

**Affiliations:** Health Behaviour Research Centre, University College London, Gower Street, London, UK; Health Psychology Research Group, Division of Psychology & Language Sciences, University College London, Gower Street, London, UK

**Keywords:** Habits, Diet, Child-feeding, Behaviour change, Intervention, Process evaluation

## Abstract

**Background:**

Forming ‘habit’ – defined as a learned process that generates automatic responses to contextual cues – has been suggested as a mechanism for behaviour maintenance, but few studies have applied habit theory to behaviour change. This study used process evaluation data, taken from a randomised controlled trial of a healthy child-feeding intervention for parents previously shown to be effective, to explore the applicability to dietary behaviour change of predictions and recommendations drawn from habit theory. The intervention supported parents in pursuing child-feeding habit goals in three domains (giving fruit and vegetables, water, healthy snacks), over four fortnightly home visits. We explored whether (a) the habit-formation model was acceptable to participants, (b) better-specified habit-formation goals yielded greater habit gains, and (c) habit gains were sustained (d) even when subsequent, new habit goals were pursued.

**Methods:**

Qualitative and quantitative data were taken from 57 parents randomised to the intervention arm, and so analyses presented here used a pre-post intervention design. Thematic analysis of post-intervention qualitative interviews evaluated acceptability, and self-reported habit goals were content-analysed. ANOVAs explored changes in habit strength, recorded at home visits and one- and two-month follow-ups, across time and goals.

**Results:**

Participants understood and engaged positively with the habit-formation approach. Although many seemingly poorly-specified habit goals were set, goal characteristics had minimal impact on habit strength, which were achieved within two weeks for all behaviours (p’s < .001), and were maintained or had increased further by the final follow-up.

**Conclusions:**

The habit-formation model appears to be an acceptable and fruitful basis for dietary behaviour change.

## Background

Dietary behaviour change interventions (BCIs) have the potential to reduce the prevalence of ill-health and premature death [1], but many have only short-term success, with effects eroding over time as old behavioural patterns are resumed [2]. Habit-formation has attracted interest as a possible mechanism for behaviour change maintenance [3]. ‘Habit’ refers to a process whereby environmental cues automatically activate an unconscious impulse to perform a behaviour that has, through repetition, become associated with those cues; ‘habitual behaviour’ denotes any action controlled by this process [4]. Habit forms through repetition of a behaviour (e.g. eating a banana) in a stable context (e.g. at breakfast [5]). This reinforces a mental context-behaviour association, to the extent that the context becomes sufficient to activate the association, which in turn triggers an impulse to perform the habitual behaviour, potentially without intention, cognitive effort or awareness [4,6,7].

Several characteristics make habit-formation relevant to behaviour change. Context-consistent performance reinforces the context-behaviour association, and so habits should be self-sustained beyond an active intervention period [8]. As habit forms, behavioural control is delegated to external cues, reducing demand on attention and memory processes [9]. Habits guide action rapidly and efficiently [10], and, in associated contexts, behaviour tends to be regulated more by habit than conscious intentions [11]. Habitual behaviours should therefore be resistant to motivation lapses that can occur after an intervention period ceases [12,13]. Commentators have called for habit-formation to be integrated into BCIs [3].

BCIs can utilise habit-formation in two ways: as an outcome, or a behaviour change technique based on promoting context-dependent repetition [3,14]. The few health-related interventions to have employed habit in both respects have targeted changes in diet or physical activity, with promising results. Participants in the ‘Ten Top Tips’ programme, which used the habit-formation model to promote healthy dietary intake and physical activity, experienced greater weight loss and habit gains at 8-week follow-up than did a no-treatment control group [15]. Weight loss correlated with habit strength gains, and was sustained at 32-week follow-up, suggesting the potential for long-term impact. Similarly, the ‘Transforming Your Life’ diet and activity habit-formation intervention resulted in greater maintenance of weight loss at six-month follow-up than a control intervention based on addressing relationships with food, body dissatisfaction and weight bias [16]. The ‘Healthy Feeding Habits’ intervention, which promoted healthy child-feeding habits among parents of children aged 2–6 years, was found to increase parental habit strength and child intake of vegetables, healthy snacks and water at 8-week follow-up [17]. Larger scale trials with longer follow-up periods are needed, one of which is ongoing [18], but these findings nonetheless support habit-formation as a strategy for lasting behaviour change, at least in energy-balance related behaviours.

Habit theory generates propositions for purposive habit development [19]. Habit-formation attempts require that people are motivated to adopt a new behaviour, translate this motivation into action, and repeat the behaviour in a specific context so that automaticity develops [20]. Some habit-formation recommendations, such as to plan the behaviour and performance context in order to repeat the behaviour at the early stages of habit development, are generic behaviour change principles. The principle of context-consistent repetition is, however, novel and unique to habit-formation: whereas some interventions prescribe a variety of goal-serving behaviours to prevent boredom (e.g. various physical activities, to achieve weight loss), habit development requires repeated performance of one behaviour in unvarying settings [5]. There is a paucity of empirical evidence around the effectiveness or acceptability of applying these theoretical principles to behaviour change interventions. Process evaluation data from habit-based interventions can offer important evidence regarding implementation of the habit-formation model [21], and provide the opportunity to test habit theory more rigorously and practically than correlational studies of existing habits [22], which currently dominate the literature on health habits [4]. To our knowledge, the only study to have explored responses to a habit-based intervention was a qualitative study of 10 participants in a pilot trial of the ‘Ten Top Tips’ weight loss intervention [23]. Participants initially found behaviour change effortful, but behaviours became increasingly automatic.

### The present study

This paper uses evidence gathered as part of the ‘Healthy Feeding Habits’ child-feeding intervention trial [17], to investigate the applicability of the habit-formation model to behaviour change practice. The present study was conducted, post-hoc, to maximise the information gleaned from the intervention trial [24]. The ‘Healthy Feeding Habits’ intervention was based on the promotion of consistent context-dependent feeding of self-chosen healthy foods from each of three food types (fruit and vegetables, healthy snacks, non-sweetened drinks). Three habit-formation goals – one for each feeding behaviour – were staggered over six weeks, with participants beginning to pursue one goal at baseline and choosing an additional habit goal at second and third visits, two and four weeks later.

Evidence from this trial showing positive effects on child dietary intake of healthy foods, and positive correlations between child intake and gains in parental feeding habit strength, has been reported elsewhere, and so behavioural and nutrition outcomes are not reported here [17]. Rather, this paper reports a secondary analysis of data from intervention recipients to explore implementability of the habit-formation model. Intervention procedures permitted four research questions to be addressed.

#### Research Question (RQ) 1: Was the habit-formation model acceptable to participants?

Qualitative post-intervention interview data allowed documentation of experiences of following dietary habit-based advice.

#### RQ2: Were ‘suboptimal’ habit-formation goals less conducive to habit gains?

Some behaviour change goals are thought to be less suited to habit-formation than others. Repetition is most likely where behavioural targets are specific, achievable and realistic [25]. Where multiple behavioural options are available (e.g. several foods can be fed to the child), it is not thought possible to form a habit for *not* doing a behaviour (e.g. not giving high-sugar cereal for breakfast [20]). Records of goals set by intervention recipients allowed us to explore whether goal characteristics determined habit gains.

#### RQ3: Were gains in habit strength sustained?

Theory predicts that habitual behaviours will remain stable over time [3], but little empirical evidence is available on habit stability. The availability of data up to 14 weeks post-baseline permitted examination of whether habit gains for newly-adopted child-feeding behaviours remained at follow-up.

#### RQ4: Were habit gains equal for goals pursued concurrently versus in isolation?

Habit-based BCIs have encouraged pursuit of several habit goals [15-17], but no study has examined whether people are disadvantaged by pursuing multiple habit goals. Habit theory offers no predictions in this respect, and several alternative hypotheses are plausible. Behaviour change can be effortful, and pursuing changes simultaneously or in quick succession may deplete finite cognitive resources. Dividing resources across multiple goals may result in reduced habit strength for goals pursued alongside other goals relative to those pursued in isolation. Alternatively, successfully negotiating the habit-formation process at an earlier stage could improve the likelihood of developing habits for subsequent behaviours, so offsetting any burden of multiple goal pursuit. We explored whether habit gains differed according to whether goals were pursued in isolation, or in addition to goals set earlier.

## Methods

### Design

Data were drawn from the ‘Healthy Feeding Habits’ intervention trial (ISRCTN09910187), which has been described more fully elsewhere [17]. Whereas the trial used a cluster-randomised controlled design, the current study used data from participants in the intervention arm only, thus representing a pre-post intervention design. Ethical approval was given by the University College London Research Ethics Committee.

### Participants

Participants were 58 parents of children aged 2–6 years, recruited from three Children’s Centres in London, using methods and eligibility criteria described elsewhere [17]. The study was advertised to parents as one that could potentially assist with feeding children a healthy diet. Parents with more than one child were told to select one ‘target child’. One participant failed to adhere to the intervention, forming two goals in one of the three target child-feeding domains and no goals in one domain, and so was removed, resulting in a final sample of 57 parents. Participants’ ages ranged from 22–56 years (mean = 35, SD = 7.7; see Table 1). Most (93.0%) were female, and just under half (47.4%) were educated to university-level. Target children’s mean age was 3 years, and half of the children (50.9%) were male.

**Table 1:** **Participant characteristics, by recruitment source**

	**All centres (n = 57)**	**Centre 1 (n = 21)**	**Centre 2 (n = 20)**	**Centre 3 (n = 16)**
***Parent Characteristics***				
Age (Years)^a^				
Mean	35.30	36.21	33.94	35.92
Range	22-56	24-56	23-48	22-49
Relationship to child				
Mother	52 (91.2%)	19 (90.5%)	19 (95.0%)	14 (87.5%)
Father	4 (7%)	2 (9.5%)	1 (5%)	1 (6.3%)
Adoptive or foster mother	1 (1.8%)	0 (0%)	0 (0%)	1 (6.3%)
Highest education level^b^				
No educational qualifications	3 (5.3%)	1 (4.8%)	2 (10.0%)	0 (0%)
GCSE or equivalent	8 (14.0%)	5 (23.8%)	2 (10.0%)	1 (6.3%)
Vocational (e.g. NVQ)	5 (8.8%)	1 (4.8%)	2 (10.0%)	2 (12.5%)
A/AS Level or equivalent	10 (17.5%)	3 (14.3%)	5 (25.0%)	2 (12.5%)
Undergraduate degree	15 (26.3%)	3 (14.3%)	5 (25.0%)	7 (43.8%)
Postgraduate degree	12 (21.1%)	6 (28.6%)	4 (20.0%)	2 (12.5%)
Living status^c^				
Home owner	17 (29.8%)	6 (28.6%)	7 (35.0%)	4 (25.0%)
Private tenant	8 (14.0%)	3 (14.3%)	2 (10.0%)	3 (18.8%)
Council tenant	29 (50.9%)	12 (57.1%)	9 (45.0%)	8 (50.0%)
Living with parent/relative	2 (3.5%)	0 (0%)	2 (10.0%)	0 (0%)
***Child Characteristics***				
Age (Years)^d^				
Mean	2.95	2.95	3.25	2.56
Range	0-6	1-6	1-5	0-5
Gender				
Female	28 (49.1%)	13 (61.9%)	8 (40.0%)	7 (43.8%)
Male	29 (50.1%)	8 (38.6%)	12 (60.0%)	9 (56.3%)
Ethnicity^e^				
White British	18 (31.6%)	10 (47.6%)	5 (25.0%)	3 (18.8%)
White other	13 (22.8%)	4 (19.0%)	5 (25.0%)	4 (25.0%)
Black Caribbean	1 (1.8%)	0 (0%)	0 (0%)	1 (6.3%)
Black African	7 (12.3%)	0 (0%)	7 (35.0%)	0 (0%)
Black other	2 (3.5%)	1 (4.8%)	1 (5.0%)	0 (0%)
Indian	1 (1.8%)	1 (4.8%)	0 (0%)	0 (0%)
Asian other	1 (1.8%)	1 (4.8%)	0 (0%)	0 (0%)
Other ethnic group	12 (21.1%)	3 (14.3%)	2 (10.0%)	7 (43.8%)

Data are frequencies and percentages of column total (excluding any missing data), unless otherwise stated. Percentages may not total 100% due to missing data.

^a^Data missing for 9 participants (2 from Centre 1, 4 from Centre 2, 3 from Centre 3).

^b^Data missing for 4 participants (2 from Centre 1; 2 from Centre 3).

^c^Data missing for 1 participant (Centre 3).

^d^Data missing for 1 participant (Centre 1).

^e^Data missing for 2 participants (1 from Centre 1, 1 from Centre 3).

### Procedure

#### Intervention

The ‘Healthy Feeding Habits’ intervention comprised four, hour-long home visits, approximately two weeks apart, conducted by trained researchers (including LM). At the first visit, parents were given an intervention booklet, which they worked through with the researcher, and which explained habit and provided habit-formation tips (e.g. identifying cues, establishing routines, planning actions and performance contexts). Three child-feeding domains were targeted: fruits and vegetables, healthy snacks and healthy drinks. The first three visits each focused upon one domain, and parents were able to choose the order in which the domains were targeted. At each visit parents were asked to specify a desired goal outcome in the chosen domain (e.g. ‘for my child to eat more vegetables’), a target behaviour and performance context (e.g. ‘to serve two vegetables with dinner’), consider ways to overcome possible barriers to achieving the habit goal (i.e. coping planning), and set a behaviour change start date. At the second and third visits, goal progress was reviewed, and parents were encouraged to continue pursuing previous goals and choose a goal in another domain. At the fourth visit, progress towards all three goals was discussed and measures taken. Parents were given self-monitoring ‘tick sheets’ to record progress between visits.

#### Data collection

Quantitative habit and behaviour measures were obtained in person at each home visit (Time 1 [T1], T2, T3, T4), and via phone calls at one and two months post-intervention (Follow-up 1 [FU1], FU2). Semi-structured interviews were conducted at the final home visit (T4), and habit goals were recorded at each home visit (T1-T4). While trial evaluation data have been reported elsewhere [17], none of the data in the present study have previously been published.

### Measures

#### Qualitative data

Interviews focused on parents’ experiences of the intervention, covering goal progress; the child’s responses; and views on the most effective elements of the intervention. Pertinent responses were recorded manually in researcher field notes. Habit goals were manually recorded by LM at T1-T3.

### Quantitative data

*Habit strength* was measured using the four-item Self-Report Behavioural Automaticity Index [26], a subscale of the Self-Report Habit Index (SRHI [27]) that has been shown to have internal reliability and convergent validity with the SRHI [26]. Items followed a stem individually tailored to the goals (e.g. ‘Serving two vegetables with my child’s dinner is something…’; ‘… I do automatically’, ‘…I do without having to consciously remember’, ‘… I do without thinking’,’… I start doing before I realise I’m doing it’; ‘strongly disagree’ [1]-‘strongly agree’ [7]). Responses were summed to generate aggregated summaries of participants’ personalised, behaviour-specific automaticity score in each child-feeding domain (all α’s. ≥ 94) [1]^a^.

Habit strength was first measured when the relevant habit goal was set. For the first habit goal pursued (‘Goal 1’), baseline habit strength scores were obtained at T1, and follow-up scores at all subsequent timepoints (T2-T4, FU1, FU2). For the second habit goal (‘Goal 2’), baseline scores were obtained at T2, with follow-ups at T3, T4, FU1, and FU2, and for the third habit goal (‘Goal 3’), baseline scores were taken at T3, with follow-ups at T4, FU1 and FU2 (see Table 2 for measurement schedule).

**Table 2:** **Habit measurement schedule**

	**Goal 1**	**Goal 2**	**Goal 3**
First visit (T1)	Baseline habit measure		
2-week visit (T2)	2 week follow-up measure	Baseline habit measure	
4-week visit (T3)	4 week follow-up measure	2 week follow-up measure	Baseline habit measure
6-week visit (T4)	6 week follow-up measure	4 week follow-up measure	2 week follow-up measure
10-weeks phone call (FU1)	10 week follow-up measure	8 week follow-up measure	6 week follow-up measure
14-weeks phone call (FU2)	14 week follow-up measure	12 week follow-up measure	10 week follow-up measure

### Analysis

To address RQ1, qualitative interview data were coded by LM using thematic analysis procedures [28], and verified in frequent discussion with BG.

For RQ2, habit goal descriptions were coded using content analysis. A coding framework was iteratively and inductively developed and applied to capture sources of variation observed within goals. Framework development and coding were undertaken iteratively by KS, in regular discussion with BG and LM. The final framework comprised five characteristics, the first four of which pertained to the target behaviour, and the fifth to the context: (1) the *number of target behaviours* specified; (2) the target *performance frequency* (possible codes: ‘once a day’, ‘twice a day or more’, ‘unclear or unspecified’); (3) whether the target behaviour was described in *absolute or relative terms* (e.g. ‘give *two* vegetables’ vs ‘give *more* vegetables’, respectively; ‘absolute’, ‘relative’, ‘combination of absolute and relative’ [applicable only to goals specifying multiple target behaviours], ‘unclear’); (4) whether an *increase or decrease in the target behaviour* was specified (‘increase in healthy behaviour’, ‘decrease in unhealthy behaviour’, ‘combination of increase and decrease’ [applicable only where multiple behaviours], ‘unclear or unspecified’); (5) the *contextual cue* (possible codes: ‘time of day’, ‘during or after a meal’, ‘during or after any other event’, ‘as a substitute for less healthy food’, ‘none specified’).

It is difficult to identify goal characteristics that are uniformly conducive to habit-formation across behaviours and contexts. Thus, for each of the five characteristics, values theoretically *least* conducive to habit development (i.e. ‘suboptimal’) were identified by BG, in accordance with theoretical principles of behaviour change and habit-formation [25] (see Table 3). Two researchers with expertise in habit-formation theory and evidence were asked to independently identify suboptimal values on each characteristic, and strong inter-rater agreement with BG was found (100% for first coder, 80% for second coder). Suboptimal values were as follows.

**Table 3:** **Characteristics of participants’ personalised habit goals and frequency of sub-optimal goals, by feeding domain**

**Characteristic**	**Observed values**	**Verbatim examples** ^**†**^	**Observed frequency**		
	(* = prejudged to be suboptimal)				
			***FV goals***	***Snack goals***	***Drink goals***
			***(n =54)***	***(n =56)***	***(n =53)***
***Characteristics of target behaviours***		***‘To give my child…’***	**n (%)**	**n (%)**	**n (%)**
1. Number of target behaviours	a. One behaviour	a. ‘… a bottle of water after school’	51 (94.4%)	52 (92.9%)	29 (54.7%)
	b. Two behaviours*	b. ‘… milk in the morning *and* more water’	3 (5.6%)	4 (7.1%)	19 (35.8%)
	c. Three behaviours*	c. ‘… juice with breakfast, water with lunch and dinner and milk at bedtime’	0 (0%)	0 (0%)	5 (9.4%)
		*Total suboptimal goals*	*3 (5.6%)*	*4 (7.0%)*	*24 (45.3%)*
2. Frequency of target behaviour	a. Once a day	a. ‘… a healthy *afternoon* snack’	49 (90.7%)	43 (76.8%)	10 (18.9%)
	b. Twice a day or more	b. ‘… one vegetable *with lunch* and one *with dinner’*	2 (3.7%)	3 (5.4%)	6 (11.3%)
	c. Unclear or unspecified*	c. ‘… extra water *during the day’*’	3 (5.6%)	10 (17.9%)	37 (69.8%)
		*Total suboptimal goals*	*3 (5.6%)*	*10 (17.9%)*	*37 (69.8%)*
3. Behaviour specified in absolute or relative terms	a. Absolute	a. ‘… *two* vegetables with dinner’	50 (92.6%)	53 (94.6%)	29 (54.7%)
	b. Relative*	b. ‘… *more* water’	0 (0%)	0 (0%)	8 (15.1%)
	c. Combination of absolute and relative *(where multiple behaviours specified)*	c. ‘…*one glass* of fruit juice a day *and more* water’	4 (7.4%)	2 (3.6%)	16 (30.2%)
	d. Unclear*	d. ‘To choose a healthy snack from the list’	0 (0%)	1 (1.8%)	0 (0%)
		*Total suboptimal goals*	*0 (0%)*	*1 (1.8%)*	*8 (15.1%)*
4. Increase or decrease in target behaviour	a. Increase in healthy behaviour	a. ‘… *more* water’	54 (100%)	52 (92.9%)	22 (41.5%)
	b. Decrease in unhealthy behaviour*	b. ‘… *less* fruit juice’	0 (0%)	0 (0%)	4 (7.5%)
	c. Combination of increase in healthy and decrease in unhealthy behaviour *(where multiple behaviours specified)*	c. ‘… *less* milk and squash *and more* water’	0 (0%)	4 (7.1%)	24 (45.3%)
	d. Unclear or unspecified*	d. ‘… one cup of milk’	0 (0%)	0 (0%)	3 (5.7%)
		*Total suboptimal goals*	*0 (0%)*	*0 (0%)*	*7 (13.2%)*
*Characteristic of context*					
5. Type of cue	a. Time of day*	a. ‘… a healthy snack *at 3 pm’*	9 (16.7%)	28 (50.0%)	1 (1.9%)
	b. During or after a meal	b. ‘… two vegetables *with dinner’*	40 (74.1%)	0 (0%)	9 (17.0%)
	c. During or after any other event	c. ‘… a bottle of water *after nursery’*	3 (5.6%)	10 (17.9%)	7 (13.2%)
	d. As a substitute for less healthy food	d. ‘… water *instead of* milk during the day’	1 (1.9%)	1 (1.8%)	7 (13.2%)
	e. None specified*	e. ‘… a cup of milk every day’	1 (1.9%)	17 (30.4%)	34 (64.2%)
		*Total suboptimal goals*	*10 (17.9%)*	*45 (80.4%)*	*35 (66.0%)*
Total ‘goal suboptimality’ score**		0 (non-suboptimal)	39 (72.2%)	9 (16.1%)	7 (13.2%)
		1	14 (25.9%)	36 (54.3%)	13 (24.5%)
		2	1 (1.9%)	9 (16.1%)	8 (15.1%)
		3	0 (0%)	2 (3.6%)	20 (37.7%)
		4	0 (0%)	0 (0%)	5 (9.4%)
		5 (most suboptimal)	0 (0%)	0 (0%)	0 (0%)

^†^Unless otherwise specified, all goals followed the words ‘to give my child…’ or equivalent. Emphases added. *Value deemed suboptimal for habit formation. **‘Suboptimality’ scores indicate the number of the five characteristics above on which the goal was deemed to be suboptimal.

#### Number of target behaviours

Mental context-behaviour associations develop more easily where a single behaviour is pursued [29]. Thus, the specification of two or more behaviours within the habit goal was deemed suboptimal.

#### Frequency of target behaviour

Specifying the frequency with a behaviour will be performed promotes contextual consistency [20]. A failure to specify the target behaviour frequency was deemed suboptimal. None of the goals observed specified multiple behaviours with varying behaviour frequencies.

#### Behaviour specified in absolute or relative terms

Specific and measurable goals better facilitate behaviour change than do vague or ambiguous goals, progress towards which cannot so easily be established [25]. Specification of the target behavioural goal in relative terms was deemed suboptimal, as were unclear or uncodeable goals. Where multiple behaviours were pursued, goals were deemed suboptimal where all behaviours were specified in relative terms, but those in which at least one behaviour was specified in absolute terms were not, as habit would at least be expected to form optimally for this behaviour.

#### Increase or decrease in target behaviour

Habit-formation requires performance of wanted behaviours, or the substitution of a wanted for an unwanted behaviour, so a specified decrease in provision of an unwanted food or drink, or goals unclear in this respect, were deemed suboptimal. Where goals specified multiple behaviours, goals were deemed suboptimal where behavioural targets were specified as decreases, were unclear, or both. Multiple-behaviour goals that specified an increase in a healthy behaviour and a decrease in an unhealthy behaviour were not deemed suboptimal, as the displacement of an undesired with a desired habit can offer an effective habit formation strategy [20].

#### Type of cue

Habit-formation is facilitated by planning performance of the target behaviour (e.g. specifying when and where) in the presence of salient, event-based cues (e.g. during mealtimes [30]). Time-based cues require conscious monitoring of the external environment and so are less conducive to habit-formation [31]. Habit goals that specified a time of day or failed to specify a performance context were deemed suboptimal. No goal was found to specify multiple cue types.

To assess whether suboptimal goals were less conducive to habit gains, goals were assigned a score of 1 for each of the five characteristics in which they were judged to be suboptimal. This produced a ‘goal suboptimality’ score of 0–5, where 5 indicated a goal deemed suboptimal on all five characteristics, and 0 a goal that was not deemed suboptimal on any characteristic. Bivariate correlations were run to estimate the relationship between goal suboptimality score and change in habit between relative baseline and two weeks post-baseline for each goal (Goal 1: T1, T2; Goal 2: T2, T3; Goal 3: T3, T4). Kolmogorov-Smirnov tests indicated that goal suboptimality scores were non-normally distributed for all goals (Goal 1: D(51) = .32, p < .001; Goal 2: D(51) = .24, p < .001; Goal 3: D(51) = .21, p < .001, respectively), and so Spearman’s rank correlation coefficients are reported. Distribution of suboptimal and non-suboptimal goals was uneven within most of the five characteristics for most goals (see Table 3). Within-category comparisons of habit gains according to goal optimality were only run for cue type, for which scores were relatively evenly distributed. A 2 (automaticity at relative baseline vs two weeks post-baseline) × 2 (suboptimal cue vs. non-suboptimal cue) mixed ANOVA assessed whether habit development for each of the three goals differed according to the context specified (suboptimal: time of day or ‘none specified’; non-suboptimal: during or after a meal or any other event, or as a substitute for less healthy food).

For RQ3, separate mixed-design ANOVAs assessed automaticity scores for each goal, with study timepoint as the within-subjects factor, and behaviour domain the between-subjects factor. Data from all available timepoints were entered for each goal (Goal 1: T1, T2, T3, T4, FU1, FU2; Goal 2: T2 [relative baseline], T3, T4, FU1, FU2; Goal 3: T3 [relative baseline], T4, FU1, FU2). Thus, automaticity was assessed over fourteen weeks for Goal 1, twelve weeks for Goal 2 and ten weeks post-intervention for Goal 3 (see Table 2). The timepoint x behaviour domain interaction was assessed to determine whether domain affected habit gains for each goal. Pairwise comparisons were run to explore significant effects, with Bonferroni corrections used to adjust for multiple comparisons. Mauchly’s test indicated that the assumption of sphericity was violated for all three analyses conducted (Goal 1: χ2(14) = 53.3, p < .001; Goal 2: χ2(9) = 47.9, p < .001; Goal 3: χ2(5) =67.9, p < .001), so degrees of freedom were corrected using Greenhouse-Geisser sphericity estimates (Goal 1: ε = .65; Goal 2: ε =0.70; Goal 3: ε = 0.59).

RQ4 was addressed via a two-factor repeated-measures ANOVA assessing habit development within the first two weeks for each goal. A two-week follow-up was chosen because habit gains are largest at the earlier stages of formation (Lally et al. [5]). The first factor was measurement timepoint, with two levels representing relative baseline and two weeks post-baseline (e.g., for Goal 2, T2 and T3). The second factor was goal number, as Goal 1 represented sole goal pursuit, but Goals 2 and 3 were pursued alongside previous goals. A timepoint x goal number interaction term examined whether habit increases observed between baseline and two weeks differed according to goal number.

Clustering was not accounted for in analyses of RQ2-4, as there was no reason to expect clustering effects given that the intervention was delivered to each participant in isolation, and habit goals were self-chosen. Indeed, observed intra-class correlation coefficients for all relevant outcomes were negligible (<.03). For all quantitative analyses, p values are presented for the sake of completion, but given the small sample and exploratory nature of the study, interpretation focuses on observed effect sizes, not statistical significance. Effect sizes were deemed noteworthy where they were at least small, as interpreted in line with Cohen’s criteria [32], whereby correlation coefficients of .10, .30 and .50, and eta-squared values of 0.02, 0.13, and 0.26, respectively represent small, medium and large effects.

## Results

### Descriptive statistics: engagement with intervention

The majority of intervention recipients provided data at all timepoints. Of 57 participants present at T1, 55 provided data at T2, 53 at T3, 50 at T4, 46 at FU1, and 45 at FU2. The most commonly chosen target behaviour for Goal 1 was fruit and vegetables (FV; 25/57; 43.9%), followed by snacks (19/57; 33.3%) and drinks (13/57; 22.8%). For Goal 2, the snack domain was most commonly chosen (29/55; 52.7%), followed by FV and drinks (both 13/55; 23.6%). For Goal 3, the drinks domain was chosen by the majority (29/53; 54.7%), followed by FV (16/53; 30.2%) and then snacks (8/53; 15.1%).

### RQ1: Was the habit-formation model acceptable to participants?

Two acceptability-related themes were extracted: favourability towards the intervention, and positive consequences of habit-formation.

#### Favourability towards the intervention

Participants found habit-formation helpful for modifying their child’s dietary intake and were glad to have taken part. For some, forming a specific feeding habit inspired broader changes in feeding routines (*“The snacks were key, as it gave us a structure to hang all the other changes off - it has really made a massive difference to routine and eating”;* Participant 16 [P16]). All participants reported understanding the concept of habit-formation and the principle of context-dependent repetition (*“It [forming habits] was very easy, I just knew in the same place at the same time each day and I remembered”;* P5).

#### Positive consequences of habit-formation

Following the intervention, many participants described their chosen behaviours as having become automatic (*“they are [now] things we do without even thinking”*; P34), and were confident about maintaining them (*“I feel like certain things are just in place… it just became part of the routine…”*; P17). For some, new habits were disrupted by changes to routines during holiday periods or major life events, such as moving home. Returning to normal environments reinstated the habitual behaviour, though for some re-establishing the habit required effort (*“[Our habits have]…gone very well despite a bit of a blip over Christmas – but we are definitely back on track now”;* P3). Habit-formation occurred at different rates, with ‘habitual’ behaviours remaining relatively effortful at the final home visit (*“I am definitely going to keep going [with these healthy behaviours, but] we aren’t there yet and I’m still thinking about what we are doing”;* P11).

### RQ2: Were ‘suboptimal’ habit-formation goals less conducive to habit gains?

Table 3 indicates the frequency with which goals were deemed, a priori, to be suboptimal for habit-formation according to each of the five coded characteristics. Characteristics of drinks goals were most commonly identified as suboptimal. Twenty-five (47.2%) drinks goals received a ‘suboptimality score’ of 3 or more, indicating that they were deemed suboptimal on at least three of the five characteristics, and only 7 (13.2%) received a score of 0. Two (3.6%) snacks goals received a score of 3 or more, and only 9 (16.1%) received a score of 0. While most fruit and vegetable (FV) goals received scores of 0 (39; 72.2%), 15 (27.8%) were deemed suboptimal on one or two characteristics.

As Table 3 shows, twenty-four (45.3%) drink goals specified two or more behavioural targets, as did 3 (5.6%) FV goals and 4 (7.1%) snack goals. The intended frequency of target behaviours was unclear or could not be coded for 37 (69.8%) drink goals, 10 (17.9%) snack goals and 3 (5.6%) FV goals. Eight (15.1%) drink goals targeted behaviours in relative rather than absolute terms, and one (1.8%) snack goal could not be coded in this respect. Few goals targeted decreases in unhealthy behaviour, or could not be coded (drinks: 7 [13.2%]; snacks: 0 [0%]; FV: 0 [0%]). A relatively high proportion of goals in each domain specified suboptimal cue types, particularly within the snacks (45 [80.4%]) and drinks (35 [66.0%]) domain (FV: 10; 17.9%).

Bivariate correlations revealed no associations between goal optimality and habit gains for Goal 1 (*r*_*s*_*=* −.06, p = .66) or Goal 2 (*r*_*s*_*=* .05, p = .70). A small, albeit statistically non-significant, negative correlation was found for Goal 3 (*r*_*s*_*=* −.15, p = .31), indicating that goals featuring more suboptimal components were associated with smaller habit gains. There was no interaction between the specification of a suboptimal cue type and time for Goal 1 (F[1, 51] = 0.48, partial eta^2^ = .009, p = .49) or Goal 2 (F[1, 51] = 0.20, partial eta^2^ = .004, p = .66). A small, but statistically non-significant, interaction effect was observed for Goal 3 (F[1,48] = 1.04, partial eta^2^ = .02, p = .31). Participants specifying either a time of day cue or no cue reported smaller habit gains over the first two weeks (mean difference [MD] =1.99, SE =0.44, p < .001) than did those specifying non-suboptimal cue types (MD =2.58, SE =0.38, p < .001). Thus, there was mixed support for a relationship between goal characteristics and habit development.

### RQ3: Were habit gains sustained?

**Figure 1:**
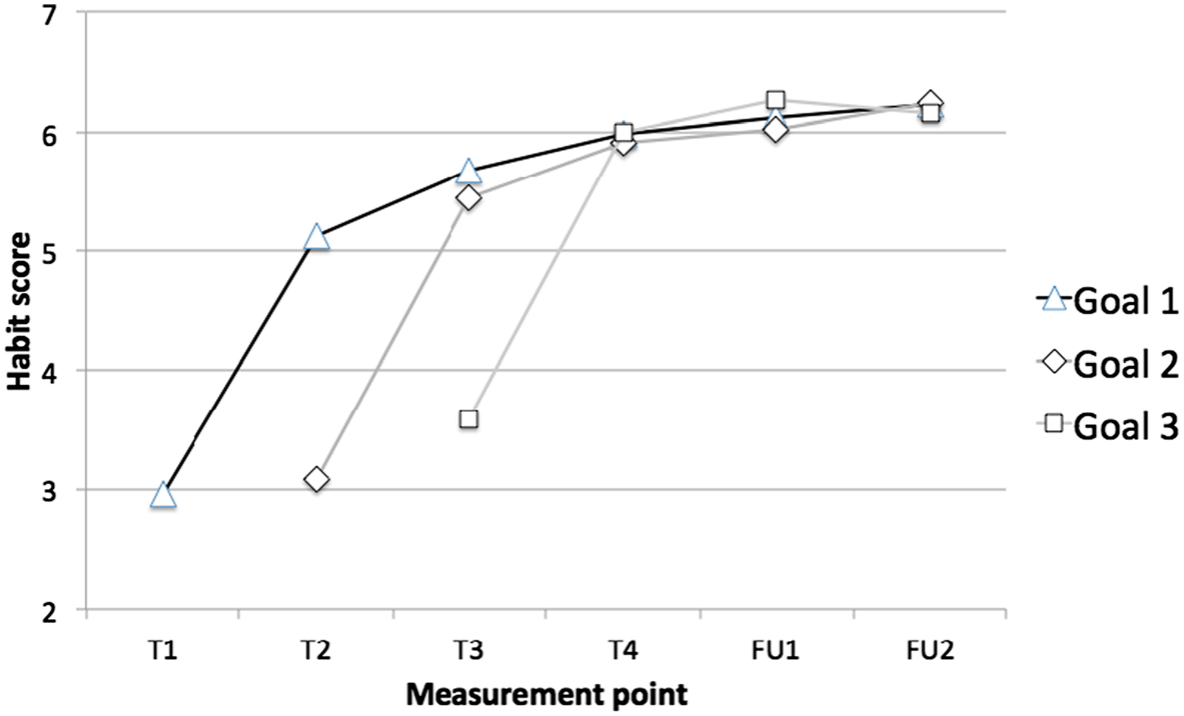
**Trajectory of mean automaticity scores over time.** Goal 1 = all goals set at T1; Goal 2 = all goals set at T2; Goal 3 = all goals set at T3.

Table 4 and Figure 1 show the trajectory of habit scores over time. For all goals, there was an effect of time on habit strength (Goal 1: F[3.26, 137.0] = 51.38, partial eta^2^ = .55, p < .001; Goal 2: F(2.78, 119.5) = 51.31, partial eta^2^ = .54, p < .001; Goal 3: F(1.76, 75.6) = 40.97, partial eta^2^ = .49, p < .001). Pairwise comparisons, conducted to investigate this effect, revealed increases in automaticity between baseline and two weeks for all goals (Goal 1: MD = 2.33, standard error [SE] = 0.29, p < .001; Goal 2: MD = 2.35, SE = 0.28, p < .001); Goal 3: MD = 2.37, SE = 0.38, p < .001). Automaticity did not change markedly between any other consecutive time points for Goal 1, but increased between two weeks and six weeks (MD = 0.85, SE = 0.19, p = .001), ten weeks (MD = 0.89, SE = 0.26, p = .02), and twelve weeks (MD = 1.01, SE = 0.26, p = .01). Automaticity did not change between any further consecutive time points for Goal 2, but did increase significantly between two and twelve weeks (MD = 0.79, SE = 0.21, p = .01). Automaticity did not change between any further time points for Goal 3 (see Table 4). Although statistically non-significant, small-to-medium timepoint x child-feeding domain interaction effects were found for each goal (Goal 1: F(6.52, 137.0) = 0.91, partial eta^2^ = .04, p = .50; Goal 2: F(5.56, 119.5) = 0.92, partial eta^2^ = .04, p = .48; Goal 3: F(3.51, 75.6) = 0.81, partial eta^2^ = .04 p = .51). Inspection of mean differences suggested that drink goals were consistently associated with larger increases in habit (range of MDs from relative baseline 2.69-4.22, maximum p = .002) than were FV (MD range 2.12-2.70, all p’s < .001) and snack goals (range 2.19-3.11, all p’s < .001). In sum, for all behaviours and goals, habit strength increased in two weeks, and either remained stable or had increased further at follow-up, and largest gains were observed for drinks goals.

**Table 4:** **Automaticity scores over time for all habit goals, by feeding domain**

**Goal number**	**Child feeding domain**		**Measurement points**					
			***T1***	***T2***	***T3***	***T4***	***FU1***	***FU2***
Goal 1	Fruits and vegetables	*n*	25	25	23	24	22	21
		*Mean (SD)*	3.02 (1.80)	5.00 (1.55)	5.42 (1.44)	5.57 (1.21)	5.82 (1.32)	5.85 (1.41)
	Healthy snacks	*n*	19	18	17	16	16	16
		*Mean (SD)*	3.13 (1.80)	5.22 (1.45)	5.76 (1.18)	6.31 (.51)	6.23 (.80)	6.45 (.63)
	Healthy drinks	*n*	13	12	11	10	9	9
		*Mean (SD)*	2.62 (1.95)	5.21 (1.73)	6.05 (1.04)	6.43 (1.03)	6.67 (.60)	6.72 (.61)
	*All domains*	*n*	*57*	*55*	*51*	*50*	*47*	*46*
		*Mean (SD)*	*2.96 (1.81)*	*5.12 (1.53)*	*5.67 (1.28)*	*5.98 (1.06)*	*6.12 (1.08)*	*6.23 (1.10)*
Goal 2	Fruits and vegetables	*n*	--	13	13	12	12	12
		*Mean (SD)*	--	3.75 (1.68)	5.58 (1.23)	5.96 (1.30)	5.98 (1.35)	6.40 (.79)
	Healthy snacks	*n*	--	29	28	26	24	24
		*Mean (SD)*	--	3.03 (1.79)	5.29 (1.58)	5.73 (1.84)	5.97 (1.20)	6.13 (.99)
	Healthy drinks	*n*	--	13	12	12	11	10
		*Mean (SD)*	--	2.52 (1.71)	5.65 (.88)	6.25 (.61)	6.18 (1.07)	6.33 (.94)
	*All domains*	*n*	*--*	*55*	*53*	*50*	*47*	*46*
		*Mean (SD)*	*--*	*3.08 (1.77)*	*5.44 (1.35)*	*5.91 (1.49)*	*6.02 (1.19)*	*6.24 (.92)*
Goal 3	Fruits and vegetables	*n*	--	--	16	14	13	13
		*Mean (SD)*	--	--	3.73 (2.04)	6.05 (1.31)	6.04 (1.24)	5.83 (1.52)
	Healthy snacks	*n*	--	--	8	8	7	6
		*Mean (SD)*	--	--	2.94 (2.01)	5.23 (1.35)	6.14 (.54)	6.04 (.62)
	Healthy drinks	*n*	--	--	29	28	27	27
		*Mean (SD)*	--	--	3.70 (2.07)	6.18 (1.35)	6.41 (1.17)	6.33 (1.21)
	*All domains*	*n*	*--*	*--*	*53*	*50*	*47*	*46*
		*Mean (SD)*	*--*	*--*	*3.59 (2.03)*	*5.99 (1.31)*	*6.27 (1.11)*	*6.15 (1.24)*

T1,…4 = Time 1,…Time 4, FU1 = Follow-up 1, FU2 = Follow-up 2. Goal 1 = goals set at T1, Goal 2 = goals set at T2, Goal 3 = goals set at T3. See Table 2 for details of measurement points. Scores range from 1–7, where 7 = strongest habit.

### RQ4: Were habit gains equal for goals pursued concurrently versus in isolation?

An effect of time on habit strength (F(1, 49) = 155.20, partial eta^2^ = .76, p < .001), indicated that, across all goals, automaticity scores increased between relative baseline and two-weeks. Goal number had an effect on habit strength (F(2, 98) = 4.57, partial eta^2^ = .079, p = .01), with automaticity scores highest for Goal 3 and lowest for Goal 1 at both relative baseline and two-week timepoints (see Table 4). There was no interaction between measurement timepoint and goal number (F(2, 98) = 0.15, partial eta^2^ = .003, p = .87). Thus, habit gains appeared similar for goals pursued in isolation (Goal 1) and those pursued alongside other goals (Goals 2 and 3).

## Discussion

This study explored the acceptability of using the habit-formation model as a basis for a dietary behaviour change intervention relating to healthy child-feeding practice. Participants randomised, as part of a controlled trial, to receive the ‘Healthy Feeding Habits’ intervention, understood the habit-formation approach and found it useful. Pre-post quantitative analyses indicated that habit strengthened immediately following the intervention, and gains were either sustained or had increased further at final follow-up. Many participants set goals that theory predicts would be ill-suited to habit-formation, and there was some evidence that doing so led to smaller habit gains. There was no apparent detrimental or beneficial effect on habit-formation of pursuing multiple goals concurrently relative to pursuing a single habit goal in isolation. Results testify to the feasibility and acceptability of basing a dietary intervention on encouragement of context-dependent performance of low-level actions, and the potential impact that this may have on the automatisation of multiple actions.

Participants appeared receptive to the habit-formation approach, and considered it easy to follow and fruitful as a behaviour change strategy. Yet, content analysis of participants’ habit-formation goals indicated considerable variation in the apparent quality of self-chosen goals, with many goals appearing theoretically suboptimal for habit-formation. Developing a habit depends on initiating and repeating a behaviour, and extensive research on goal-setting has revealed that the goals most effective for instigating behaviour change are specific and measurable [25]. Additionally, the mental context-behaviour association that underpins the habit process is best fostered by careful planning of a specific action in the presence of salient, event-based cues located within existing routines [30,31]. However, many participants’ habit goals were vague, specified multiple behaviours, failed to identify a context for performance, or used time-based cues, which require continuous environmental monitoring and so are ill-suited to automatisation [31]. Our data do not reveal why such variation in goal quality was observed. Post-hoc discussions with those who delivered the intervention revealed that, in the absence of guidance on optimal goal-setting, they felt ill-equipped to challenge parents who chose targets that conflicted with theoretical predictions. Interventions might benefit from offering clear guidance on both the importance of habit-formation and the characteristics of goals theoretically most conducive to habit development, and ensuring that staff are trained not only in intervention delivery, but also in the theory and evidence base of the intervention.

Goals set at the third timepoint which specified either a time-based cue or no cue tended to be associated with smaller habit gains over the first two weeks of the habit-formation attempt. This supports predictions that situation-specific goals better aid development of mental cue-behaviour associations [30], and time cues may be less conducive to habit-formation because responding to them requires external monitoring [31]. Yet, habit goals featuring time-based cues or no cues were associated with significant habit gains, and no relationship was found between characteristics of the first two goals set and resultant habit strength. These findings suggest that variation in properties of the behavioural target and context specified within the goal do not preclude habit-formation. There are several potential explanations for the discrepancy between our findings and goal-setting theory and evidence [25]. Participants may have revised their goals so as to be more conducive to habit-formation after recording them, or recorded goals may have failed to articulate true plans. Alternatively, it is possible that in this intervention, goal-setting had little impact on habit change. Other techniques, such as the provision of information on the importance of healthy child-feeding practice and suggested strategies for feeding children, or the support provided by multiple one-to-one home visits, may have caused most change in behaviour, largely independently of the goals set. Incidental subsequent repetition in a stable context may have strengthened habit. Indeed, some intervention studies have reported habit strength increases even where context-dependent repetition was not reported as an intervention component [33,34]. No study has yet compared the effectiveness of interventions that explicitly promote context-dependent repetition with matched interventions that do not [4], so it is difficult to identify whether explicit advice on forming habit is required for habit to strengthen. Another possibility is that our operationalisation of goal quality was inadequate. Nonetheless, given both the small effect of cue type on habit gains observed in our data, and the weight of extant evidence around the importance of goal-setting for behaviour change [20], it would seem prudent for habit-based interventions to provide clear guidance on setting behaviourally and contextually specific action plans.

Intervention recipients achieved sustained increases in habit strength following exposure to the intervention. Habit strengthened most rapidly and extensively within the first two weeks, and either remained stable or had strengthened further at the final follow-up. While gains were more pronounced for drinks than for fruit and vegetable or snack goals, increases in habit were observed across all three feeding goals. Habit gains were equal for goals pursued in isolation at the start of the intervention period and those pursued concurrently with other goals at later stages of the intervention. Together, these results suggest that interventions may feasibly achieve sizeable and rapid progress towards multiple behavioural habit targets. These data require qualification, however. Our intervention promoted the adoption of simple behaviours, which tentative evidence suggests may become habitual more quickly than complex behaviours [5]. More work is needed to investigate the limits of people’s ability to manage concurrent pursuit of complex target behaviours. It may also perhaps be easier to pursue goals within the same behavioural domains than completely distinct areas (e.g. dietary change and smoking cessation). Additionally, within the intervention studied here, habit goals were set on three occasions, each two weeks apart. Given that the most pronounced habit gains took place in the first two weeks, participants may not have been actively continuing to pursue habit goals set at earlier points, and so we may not have captured concurrent habit pursuit, but rather the adoption of new goals where previous goals have been achieved. A more rigorous analysis of concurrent habit goal pursuit would require an experimental design in which participants are randomized to form either one, two, three or more habits concurrently, across a variety of behavioural domains. While our findings support the notion that habit strength may persist over time, further research with longer follow-up periods is needed to explore habit maintenance [4]. One study observed decreases in automaticity at six-month follow-up among recipients of a habit-based flossing intervention [35], suggesting that habits may decay over the long-term [8].

Limitations of this study must be acknowledged. It is unclear to what extent the successful implementation of the habit-formation model within the Healthy Feeding Habits intervention may be replicated across samples and settings. Some participants felt that the home visits with a trained researcher were integral to habit-formation, but such a delivery method is unlikely to be feasible for most public health interventions. Further work could usefully test the acceptability of self-administering habit-formation advice. Qualitative data were collected by the same researchers who delivered the intervention, and analysed by one of these researchers. Although it was made clear that all feedback, positive or negative, was welcome to aid development and improvements to the intervention, participants may have felt pressured to provide positive and socially desirable comments. No measures were taken of the frequency with which goal behaviours were enacted, and so associations between initial repetitions, habit-formation, and consequent behavioural stability, as predicted by habit theory [4], could not be documented. While ‘tick-sheets’ were available to us on which parents recorded whether the target behaviour had been performed on a given day, they were used primarily for intervention purposes, to facilitate self-monitoring, and use of ticksheets was not compulsory. Consequently, we deemed these to be unreliable indicators of performance frequency. Indeed, the post-hoc nature of the study imposed constraints on analyses, as the measures used were neither designed nor collected for the purposes for which we used them. Thus, the time points at which data were collected were not comparable across all habit-formation goals. The small available sample, while adequate for detecting expected intervention effects on behaviour [17], may not have conferred sufficient power on the present, secondary analyses. We allowed for this by interpreting findings on the basis of effect size, rather than statistical significance, but it remains possible that the observed effects were unreliable due to the small sample. Future intervention trials should be designed, a priori, to yield information on the processes through which the intervention has its effects, and likely responses to future iterations of the intervention.

## Conclusions

Our study provides valuable process evaluation data that attests to the acceptability of using the habit-formation model as a basis for a behaviour change intervention. Longer-term data are needed to test the potential for newly-formed habits to maintain behaviour, and more evidence is required to explore the extent to which goal-setting impacts on habit-formation in the real world. Nonetheless, our results suggest that theory-based principles of habit-formation offer an acceptable and fruitful basis for changing dietary behaviour.

## Consent

Written informed consent was obtained from childrens' parents/guardians for the publication of this report.

## Endnote

^a^Two sets of habit strength measures were taken within the Healthy Feeding Habits intervention trial [17]. Participants in the control and intervention arms completed habit measures that used generic wording for each domain (e.g. ‘*giving my child five fruits and vegetables per day* is something I do automatically’). Participants in the intervention arm also completed habit measures tailored to their personal goals (e.g. ‘*giving my child a bottle of water after school* is something I do automatically’). The present study focuses on the latter measures only, which have not previously been reported or analysed.

## Abbreviations

ANOVA: Analysis of variance
BCI: Behaviour change intervention
FU: Follow-up
FV: Fruit and vegetables
MD: Mean difference
RQ: Research question
SD: Standard deviation
SE: Standard error
SRHI: Self-Report Habit Index
